# Cognitive behavioural therapy in comparison to treatment as usual in young adults at high risk of developing bipolar disorder (Bipolar At Risk): a randomised controlled trial to investigate the efficacy of a treatment approach targeted at key appraisal change: Bipolar At Risk Trial II (BART II)

**DOI:** 10.1186/s12888-025-06973-3

**Published:** 2025-07-01

**Authors:** Sophie Parker, Lydia Pearson, Rebekah Carney, Richard P. Bentall, Matthew R. Broome, Emma Cernis, Timothy Clarke, Steven Jones, Katherine Moran, Jonathan Wilson, Isabel Coleman, Catherine Hewitt, Wendy Jones, Heather Law, Sarah Peters, Gemma Shields, David Shiers, Luke Strachan, Anton Strong, Judith Watson, Chris J. Sutton

**Affiliations:** 1 Youth Mental Health Research Unit, Greater Manchester Mental Health NHS Foundation Trust, Manchester, M25 3BL UK; 2 Division of Psychology and Mental Health, Faculty of Biology, Medicine & Health, University of Manchester, Manchester, M13 9PL UK; 3 School of Psychology, Faculty of Science, University of Sheffield, Sheffield, UK; 4 Institute for Mental Health, School of Psychology, University of Birmingham, Birmingham, UK; 5 Birmingham Women’s and Children’s NHS Foundation Trust, Birmingham, UK; 6 Children and Young People’s Mental Health Service, Norfolk and Suffolk NHS Foundation Trust, Norwich, UK; 7 Spectrum Centre for Mental Health, Lancaster University, Lancaster, UK; 8 York Trials Unit, University of York, York, UK; 9 Equality Diversity & Inclusivity Research Unit, Greater Manchester Mental Health NHS Foundation Trust, Manchester, M25 3BL UK; 10 School of Medicine, Keele University, Staffordshire, UK; 11 Lancashire Clinical Trials Unit, University of Central Lancashire, Preston, UK

**Keywords:** Bipolar at risk, Bipolar disorder, Mood swings, Early intervention, Early detection, Cognitive behavioural therapy, Randomised controlled trial, Prevention, Psychological therapy, Youth mental health

## Abstract

**Background:**

Research has demonstrated the ability to identify and treat individuals at high risk of developing psychosis. It is possible to use a similar strategy to identify people who have an emergent risk of bipolar disorder (BD). Interventions during the early phase may improve outcomes and reduce risk of transition. Criteria have been established to identify individuals considered to be at high risk for developing BD, also known as Bipolar At Risk (BAR). Offering a psychological intervention may provide the possibility of prevention. Evaluating efficacy and the mechanisms by which this treatment works is now required.

**Methods:**

A multicentre, rater-masked randomised controlled trial with two parallel arms will compare cognitive behaviour therapy (CBT) for young people meeting BAR criteria (CBT_BAR_) + Treatment as Usual (TAU) vs. TAU alone. Participants will be recruited from five National Health Service (NHS) sites in the UK. Outcome and mediational variables will be collected at baseline, 17-weeks (in treatment), 27-weeks (post-CBT_BAR_ /TAU), and 52-weeks. Qualitative work will examine the perceived mechanisms of change and implementation of CBT_BAR_ in the NHS.

**Discussion:**

Our efficacy hypotheses are CBT_BAR_ + TAU (compared to TAU alone) will lead to improvement in mood swings, a reduction in the likelihood of transition to BD, and improvements to functioning and quality of life. Our mechanistic hypothesis is CBT_BAR_ + TAU causes improvement in mood swings due to the reduction of extreme positive and negative appraisals of internal states which in turn improves subsequent behaviours used to control mood and then internal states. Our trial will explore the perceived mechanism of change via this novel intervention (CBT_BAR_) and if the approach can be implemented within current services in the UK.

**Trial registration/Status:**

The trial protocol is registered with ISRCTN (ISRCTN13363197, registered on 25th January 2023). Recruitment started in February 2023 and is ongoing.

**Supplementary Information:**

The online version contains supplementary material available at 10.1186/s12888-025-06973-3.

## Background

An estimated 1–3% of the population are affected by Bipolar Disorder (BD) [1, 2], which poses particular risks for young people. At least 25–50% of people with BD attempt suicide at least once [3], with the World Health Organisation identifying BD as a major cause of mortality and morbidity in youth (aged 10–24) [4]. With an average duration of untreated illness (DUI) of 6–10 years [5], those with adolescent onset have prolonged DUI [6] leading to increased mood episodes and elevated suicide risk [7]. This is particularly significant given that BD has the highest suicide rates among psychiatric diagnoses [8], with deaths often occurring in those with longer illness duration [9]; therefore, there is a unique opportunity for early intervention to change this trajectory.

The James Lind Alliance identified priorities for those with BD including rapid access to diagnostic assessments, developing effective talking therapies such as cognitive behaviour therapy (CBT) and individually tailored treatments [10]. Early interventions in psychosis services show health and economic benefits [11] and youth service models propose to widen intake criteria to encompass BD and those at risk of BD with the aim of reducing symptoms and risk of progression to more severe illness [12]. Extending early intervention and early detection services to include BD could yield £35 m savings in the UK [13], particularly for those meeting bipolar at-risk criteria (BAR) [14, 15], and who are help seeking, distressed National Health Service (NHS) patients.

Early detection of BD has focused on familial risk [16–19] and identification of state-trait factors [12]. Detection of those at risk for BD is possible using standardised criteria. BAR criteria [14, 15] consist of youth (16–25) experiencing distressing high mood; and/or high and low mood swings; and/or a first degree relative with BD plus depressed mood. This has predictive validity, can be reliably assessed (in an NHS context), holds clinical utility and is suitable for numbers needed to screen [5]. Most people meeting BAR criteria present with depressed mood (often atypical depression) [20] and mood swings which are generally poorly recognised and misdiagnosed. This leads to inappropriate treatments e.g. antidepressants which can induce mania [21], or psychological treatments for unipolar depression that do not target modifiable risk factors for atypical depression or mood swings. BAR individuals are 100 times more likely to convert to first-episode mania than the general population, and 20 times more likely than those with unipolar depression [22, 23], representing a unique chance to intervene.

Minimal evidence exists about effective treatment options for those meeting BAR criteria. National Institute for Health and Care Excellence (NICE) guidelines recommend offering people with BD psychological interventions (CBT) [24]. For children and young people, pharmacological treatment is only recommended when symptoms are severe [24]. Treatment access is difficult and lengthy and duration of untreated illness is linked with poor outcomes [25]. Yet, it is possible to deliver treatments in routine services which can have beneficial effects [26]. Meta-analyses report the efficacy of CBT to reduce relapse and improve symptoms of depression, mania, and functioning in BD [27–31] and that it is cost-effective when compared with TAU [32]. A recent randomised controlled trial (RCT) found CBT significantly improved outcomes in recent onset BD [33], and studies of psychological therapies for young people with BD report benefits of CBT [34]. NICE do not yet recommend CBT as a treatment for BAR individuals due to no consensus regarding early screening and lack of quality trials [24]. A rigorous RCT is needed to evaluate the efficacy of CBT to reduce distressing mood swings and understand to what extent CBT reduces mechanisms central to a model of mood swings [35]. A review assessing pharmacological interventions for BAR found a lack of high-quality research on preventative treatments [36], so could not conclude whether pharmacological approaches are beneficial or harmful. Coupled with potential safety considerations, psychological interventions might have an advantage over pharmacological interventions [37], particularly since pharmacological treatments are not always successful for established BD.

To address this evidence gap, we conducted a feasibility trial (Bipolar At Risk Trial (BART), conducted from 2015 to 2018), funded by the National Institute for Health Research (NIHR) Research for Patient Benefit (RfPB) programme (PB‐PG‐1013‐32,044). The results highlighted the 76 participants meeting BAR criteria were help-seeking, distressed NHS patients, with complex and co-morbid difficulties, and in need of specialist intervention [38]. These findings are similar to those reported by Bechdolf [15] who evidenced high levels of unemployment, suicide attempts and Axis I disorders. Participants’ treatment pathways demonstrated the breadth of treatments and services that people had accessed for help. The BART feasibility trial showed the CBT intervention was safe and acceptable, and signalled positive therapeutic effects. It was a single-site study completed in Greater Manchester; considered a large and diverse city but may not be fully representative of the wider BAR population. A larger, multi-site RCT is needed to expand the evidence base of effective treatments for NHS patients and understand any potential mechanisms by which this treatment may improve clinical outcomes.

### Aims/Objectives

The overall aim is to conduct a two-arm multicentre, rater-masked, randomised controlled trial comparing CBT_BAR_ plus TAU vs. TAU alone for BAR individuals to evaluate the efficacy of a specific intervention (CBT_BAR_). We also aim to investigate how CBT_BAR_ impacts on the pathway between key psychological processes and mood swings (See Table 1. Aims, Objectives and Outcomes). The overarching research questions are:

**Table 1:**
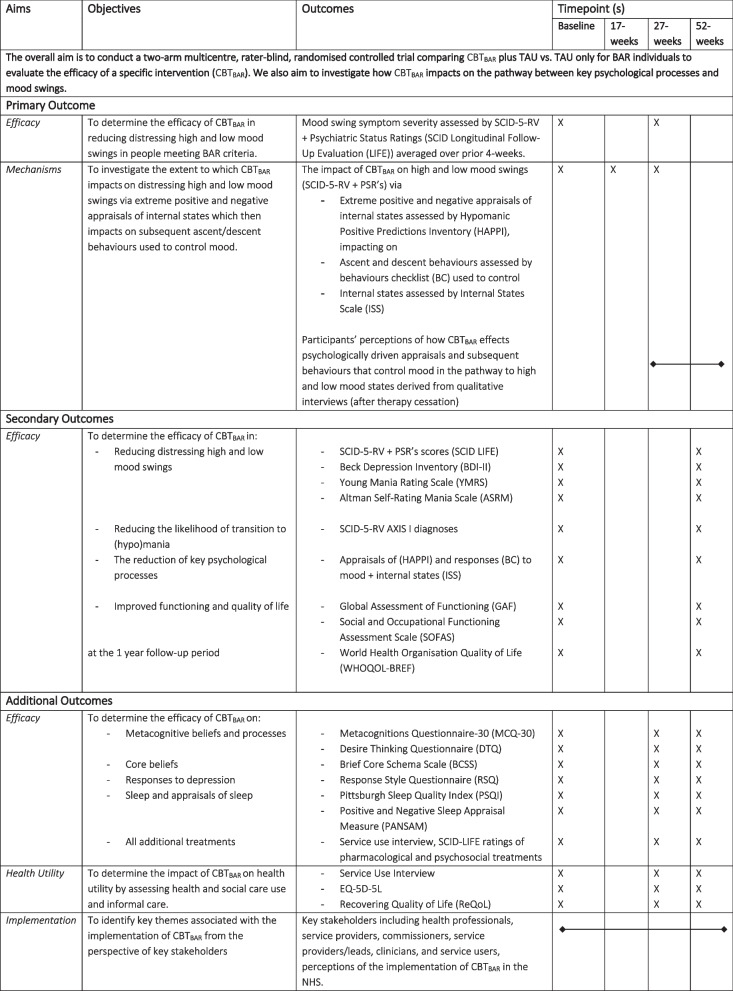
Aims, objectives and outcomes

1. To what extent is CBT_BAR_ (a psychological therapy) effective in reducing distressing mood swings compared with TAU for BAR individuals? (at 27-weeks)
2. To investigate the extent to which CBT_BAR_ impacts on the pathway between key psychological processes and mood swings (at 17-weeks).
3. What are the perceptions of patients and heath care professionals regarding the implementation of therapy in NHS services?

## Methods

### Trial design and flow chart

**Fig. 1:**
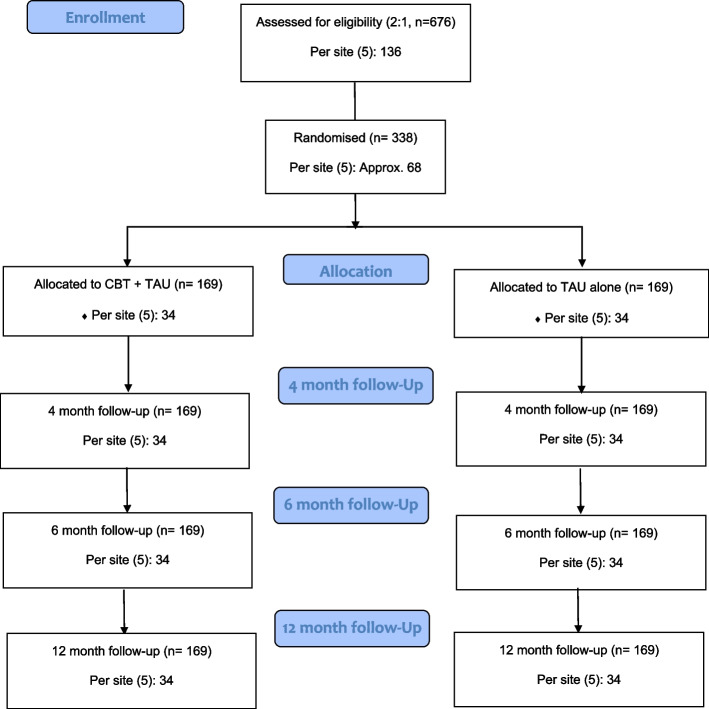
CONSORT 2010 flow diagram: BART II

The BART II trial is a rater-masked, randomised controlled trial (RCT) with two parallel arms comparing a psychological intervention (CBT_BAR_) plus Treatment As Usual (TAU) to TAU alone (control condition). There will be two nested components: 1) a qualitative sub-study to understand the perceived mechanisms of change for participants offered the CBT_BAR_, as well as the implementation of CBT_BAR_ in NHS services; and 2) Inclusivity Workstream. Outcome and mediational variables will be collected at baseline, 17-weeks (during treatment window), 27-weeks (after therapy cessation) and 52-weeks. See Fig. 1 for the CONSORT flow diagram.

The trial was prospectively registered on the ISRCTN registry (ISRCTN13363197) prior to recruitment commencing. The study was funded by the Efficacy and Mechanism Evaluation (EME) Programme, an MRC and NIHR partnership (NIHR132622). All work has been developed and will be reported in line with CONSORT Extension to Randomised Controlled Trials (http://www.equator-network.org/reporting-guidelines/consort/), SPIRIT guidelines (http://www.spirit-statement.org/), and the TIDieR checklist (http://www.bmj.com/content/348/bmj.g1687) (see Supplementary Data). An independent Trial Steering Committee (TSC), Data Monitoring and Ethics Committee (DMEC) and multiple lived-experience advisory groups have been set up to provide guidance and oversight to the trial.

### Study setting

The trial will be conducted in five community-based NHS foundation trusts (UK): Greater Manchester Mental Health NHS Foundation Trust (GMMH), Lancashire and South Cumbria NHS Foundation Trust (LSCFT), Sheffield Health and Social Care NHS Foundation Trust (SHSC), Birmingham Women’s and Children’s NHS Foundation Trust (BWC), and Norfolk and Suffolk NHS Foundation Trust (NSFT).

### Participants

The inclusion/exclusion criteria are:

#### Inclusion criteria

i. 16–25 years old,
ii. Help seeking,
iii. Able to provide written, informed consent, and
iv. Meets criteria for at least one BAR group within the last 12-months (See Table 2).

**Table 2:** Bipolar At Risk (BAR) groups (Adapted from Bechdolf [14] with modified group classifications)

BAR group name	Criteria as assessed by the Structured Clinical Interview for DSM-5 Research Version (SCID-5-RV)	Duration of symptoms
1. Subthreshold mania	Abnormally and persistently elevated, expansive or irritable mood and at least two criteria from the symptom list (three if mood is irritable): Inflated self-esteem/grandiosity; Decreased need for sleep; More talkative than usual or pressure to keep talking; Flight of ideas or racing thoughts; Distractibility; Increased goal-directed activity or psychomotor agitation; excessive involvement in activities that have a high potential for painful consequences	At least two consecutive days but < 7
2. Depression + cyclothymic features	Depressed mood or loss of interest/pleasure for at least one week and at least two criteria from the symptom list: Significant weight loss; Insomnia or hypersomnia nearly every day; Psychomotor retardation or agitation; Fatigue or loss of energy; Feelings of worthlessness or excessive/inappropriate guilt; Diminished ability to think or concentrate; Recurrent thoughts of death or suicidal ideation *Plus* *S*ubthreshold mania symptoms as described in group 1 (but see “Duration of symptoms”)	Depression: at least one week Subthreshold mania symptoms: four hours within 24-h period, on at least four cumulative, lifetime days
3. Depression + genetic risk	Depression symptoms as described in group 2 *Plus* First-degree relative with BD	Depression: at least one week

^*^When a participant meets more than one BAR group at baseline, the BAR group for randomisation input will be decided using a hierarchical rule, with BAR Group 1 entered when met, BAR Group 2 entered when met on own or in combination with BAR Group 3, and BAR Group 3 only entered when met alone

#### Exclusion criteria

i. History of a treated/untreated manic episode or psychosis of 1-week duration or longer,
ii. Treatment with a mood stabiliser for longer than 6 weeks or antipsychotic for 3 weeks (that evidence exclusion on point above or at the time of the assessment whereby at-risk status cannot be confirmed),
iii. Organic brain disorder,
iv. Inability to complete assessments due to language barriers,
v. Inpatient/acute psychiatric care needed, or primary substance abuse/dependency.

To ensure the study results are readily translatable within the current NHS, recruitment will involve outreach to a variety of services including children and young people’s mental health services, early intervention and detection teams (EDIT/EIT), Community Mental Health Teams (CMHT), Improving Access to Psychological Therapies (IAPT), primary care psychology services, GPs, schools, university health services, other youth services and the voluntary sector. Teams will be provided with study materials and be asked to identify potentially eligible individuals to refer to the research team. Referrers will discuss the study and obtain consent-to-contact for researchers who will then provide the individual with information to enable them to provide informed consent. Potential participants will be screened for potential eligibility before informed consent is taken. After written informed consent is obtained, BAR status will be established using the SCID-5-RV [39] and eligibility confirmed with the chief investigator or their delegate. Participants will be informed that they can withdraw from the trial at any point, without giving a reason and without it affecting their care.

### Inclusivity workstream

Data demonstrates significant health inequalities for people from UK ethnic minority groups, particularly in mental health treatment access/offer [40] and trial recruitment [41]. This is especially true of access to treatments which are preventative and early in the care pathway [42] versus differential higher rates within acute and secure pathways e.g. inpatient care and forensic services [43]. As there are no meaningful differences between ethnic groups in terms of the likelihood of screening positive for bipolar disorder [44] it is paramount that specific efforts to reach out to UK ethnic minority participants are required.

The BART II inclusivity workstream aims to deepen understanding of the needs of diverse communities we are seeking to help by; identifying underserved groups, assessing research acceptability, understanding barriers, developing solutions, and creating necessary resources to ensure the BARTII research methods and outcomes remain meaningful for all eligible populations.

This workstream will gather referral data, evaluate a co-produced animation aimed at enhancing recruitment of ethnic minority youth (16–25) via a Study Within A Trial (SWAT 220; SWAT220 Sophie Parker, Chris Sutton, Parise Carmichael-Murphy, Lydia Pearson, Heather Law, Sarah Rhodes, Eleftheria Patetsini, Luke Strachan, Izzy Coleman (2022 JUN 16 2222).pdf), explore service access experiences, and develop inclusive recruitment recommendations. A Patient and Public Involvement group comprising UK ethnic minority individuals will provide essential insight and oversight.

### Randomisation and masking

Following informed consent and entry to the trial, participants will be randomly allocated to one of two trial arms. Randomisation will be independent and concealed, using permuted stratified blocks (by site (5-levels) and BAR group (3-levels)) via a web-based system at York Trials Unit (YTU). Stata v18.0 [45] was used to generate the allocation schedules. Researchers will enter participant’s details into the randomisation system and the outcome communicated to the chief investigator, trial management and administrators. Participants, their GP, and referrers will be informed about the allocation via letter.

Assessor’s will be masked to treatment condition. Masking will be maintained using various measures including separate offices for therapists and research assistants, reminders about masking, protocols for message taking, and data security using passwords and encryption. Letters to participants and clinicians will contain a standardised statement about the need to maintain the masking process. Unmasking will be recorded, and where possible an independent assessor with whom the masking has not been broken will complete follow-ups.

### Study arms

#### Intervention arm: the CBT_BAR_ intervention plus TAU

The CBT_BAR _intervention uses a model [46], which draws on a cognitive model of mood swings (Integrative Cognitive Model; ICM) [47]. Appraisals of internal states are central to the ICM, and often have multiple extreme, personalised and conflicting meanings (positive and negative). These extreme appraisals give rise to competing strategies to control internal states resulting in ascent and/or descent behaviours (dependent on the goal in mind), which cause shifts in mood states. CBT_BAR_ is also informed by a cognitive model tested for young people at risk of psychosis [48] where intrusions are often interpreted as threatening. As such, safety-seeking behaviours are employed serving to maintain difficulties. Interpretations are driven by life experiences and beliefs and knowledge about the self, the world and others. CBT_BAR_ formulations guide interventions aimed at reducing distressing mood swings by (1) changing appraisals, (2) reducing unhelpful coping strategies, and (3) providing increased awareness of mood states and associated behaviours. Targeting appraisals of mood states and unhelpful coping behaviours as key mechanisms, reduces escalating mood swings, lowering the likelihood of transition to bipolar disorder and improving recovery and quality of life.

The CBT_BAR_ intervention [49] components broadly fall under three categories: 1. Core principles and values (trusting relationship, validation and normalising experiences, and collaborative goal setting); 2. Cognitive change strategies targeting appraisals; 3. Behavioural strategies aimed at modifying responses. It is delivered via 26 sessions within a 6-month intervention window and treatment follows four stages: assessment and engagement, change strategy phase, longitudinal formulation phase and consolidation phase. Sessions are flexible, allowing for in person or online delivery by trial therapists (Clinical Psychologists and Psychological Therapists).

### Control arm: TAU only

The control condition is TAU plus follow-up. Referrers will be instructed to not withhold treatment. TAU may include standard psychiatric care, psychological and vocational interventions from various agencies (although, in our experience, provision for this population is poor). Access to services includes IAPT, Children and Adolescent Mental Health Services (CAMHS), Primary Care, Early Intervention Teams and CMHTs. CBT_BAR_differs from standard NHS treatment for young people with mood swings as highlighted in our feasibility trial [38]. All routine or additional treatments in both conditions will be monitored using a Treatment Documentation Sheet and specific treatments (anti-depressant and psychotherapy treatment) monitored within the LIFE [50] assessment tool.

TAU represents an enhancement over routine care since symptoms of mania will be detected earlier than in usual practice and appropriate treatment referrals made. Participation in assessments may reduce the (frequently high) number of contacts required to receive appropriate treatment for BD. Assessments may identify untreated BD and any risks to self or others that require immediate action. TAU alone will not include liaison with a clinical team, except where risk is concerned.

### Assessments and outcomes

**Fig. 2:**
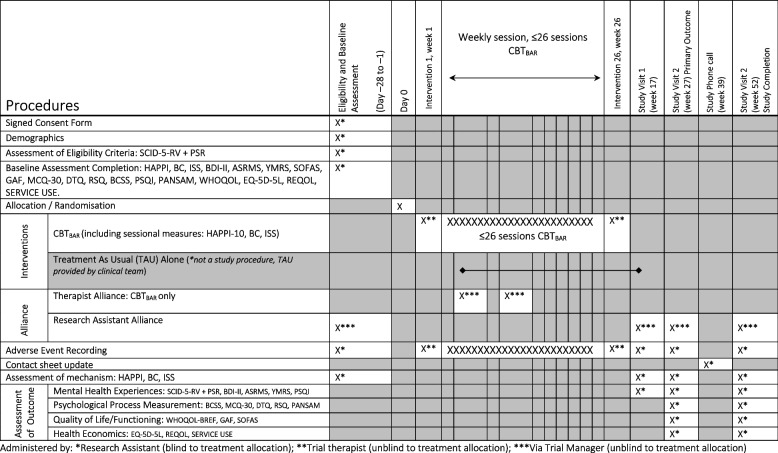
Schedule of enrolment, intervention and assessments

Assessors masked to treatment group will collect outcome variables at baseline, 17-weeks, 27-weeks (after therapy cessation/TAU), and 52-weeks (see Fig. 2 for schedule of enrolment, intervention and assessments). Assessments will be via semi-structured interviews and self-report questionnaires. Participants will be compensated for the time taken at each data collection point (£20). Contact will be made at 39-weeks to promote retention and re-confirm contact details. Participants will have flexibility to choose when and where they would like to be seen e.g. in non-stigmatising settings such as their home, youth centres, colleges, or primary care centres. Measures to be collected are listed below.

#### Demographic information

Demographic information will be collected at baseline including sex, gender, age, sexual orientation, and ethnicity. An additional demographics form capturing education, employment, marital status, living arrangements, receipt of benefits and criminal convictions will be completed at baseline and then checked at the 27- and 52-week follow-ups for changes.

#### Co-primary mood outcome measure

The SCID-5-RV with Psychiatric Status Ratings (PSR), which incorporates the SCID Longitudinal Follow-Up Evaluation (LIFE) [50], is used to assess the severity of depressive and manic symptoms over the prior 4 weeks, with measurements taken at the 27-week timepoint. Two scores are provided, depression (on which the sample size is primarily based), and mania.

#### Secondary outcomes

Appraisals of and responses to mood (hypothesised mechanisms).

The following self-report measures will assess key components of the predicted psychological pathways to mood swings:

1. Hypomanic Positive Predictions Inventory (HAPPI) [51] assesses multiple, extreme, and personalised appraisals about high and low mood.
2. Behaviours Checklist (BC) [52] measures ascent and descent behaviours triggered by extreme positive and negative appraisals about internal states.
3. Internal States Scale (ISS) [53] assesses internal mood states and has four subscales: Activation, Depression, Well-Being, and Conflict.

### Secondary mood outcome measures

1. Beck Depression Inventory 2nd Edition (BDI-II) [54] assesses severity of depression.
2. Altman Self-Rating Mania Scale (ASRM) [55] assesses the self-reported presence of and/or severity of mania symptoms.
3. Young Mania Rating Scale (YMRS) [56] assesses severity of mania.

### Additional Secondary Outcome Measures

1. The Global Assessment of Functioning scale (GAF) [57] measures social, occupational, and psychological functioning.
2. Social and Occupational Functioning Assessment Scale (SOFAS) [58] is a global rating of current functioning.
3. World Health Organisation Quality of Life (WHOQOL-BREF) [59] will be administered to assess quality of life.
4. Metacognitions Questionnaire–30 (MCQ-30) [60] is a self-report measure that assesses metacognitive beliefs related to worry and intrusive thoughts.
5. Desire Thinking Questionnaire (DTQ) [61] measures metacognitions about desire thinking, which is the verbal and imaginal elaboration of a desired target.
6. Brief Core Schema Scale (BCSS) [62] measures core beliefs about the self and others and has four subscales (negative-self, positive-self, negative-other, and positive-other).
7. Response Style Questionnaire (RSQ) [63] is a self-report measure of stable trail-like behaviours observed in response to depression.
8. Pittsburgh Sleep Quality Index (PSQI) [64] assesses sleep quality.
9. Positive and Negative Sleep Appraisal Measure (PANSAM) [65] assesses for extreme positive and negative sleep appraisals with regards to sleeping more or less than usual.

### Health economics measures

Measures collected to inform health economic analysis include:

- EQ-5D-5L [66] measure of health status is a generic measure for describing and valuing health on 5 domains: Mobility, Self-Care, Usual Activities, Pain/Discomfort, and Anxiety/Depression, and overall health.
- Recovering Quality of Life (ReQoL-10) [67] measures domains that are relevant to the recovery of people with mental health difficulties.
- Service use questionnaire adapted from previous trials, collecting data on inpatient, outpatient, accident and emergency, primary, community and social care use.

### Therapy session measures

For those in the intervention arm, a 10-item HAPPI is derived from the full measure [51], incorporating a mixture of positive and negative beliefs given the evidence that the co-occurrence of these beliefs is highly predictive of mood swings [35]. This 10-item self-report measure is completed at each session alongside the ISS [53] and the Beck Depression Inventory – Fast Screen (BDI-FS) [68]. The measures are used to guide interventions and track change over time.

### Measures of alliance, engagement, and adherence

All participants will complete, a Facilitative Alliance Inventory (FAI) [69] at baseline, 27-week and 52-week follow-ups to assess alliance with the researchers. For those in the intervention arm, therapeutic alliance will be assessed after session 4 and 10 with the California Psychotherapy Alliance Scale (CALPAS) [70] completed by both the therapist and participant. Therapists will record the number of sessions attended, duration of the sessions, and the session record form as a measure of adherence.

### Nested qualitative study

The qualitative component explores perceived mechanisms of change through individual semi-structured interviews with a maximum variance sample of participants selected for their varying therapeutic alliance scores, post-intervention mood experiences, and appraisal changes. We will also examine CBT_BAR_ implementation in NHS services through individual semi-structured interviews with key stakeholders (e.g., family members, healthcare professionals, and service providers) across multiple sites.

### Data management

Study data are collected using paper Case Report Forms and transferred to a secure, web-based software platform (REDCap) [71, 72] hosted by York Trials Unit, who provide data management and oversight. Access to the study interface will be restricted to named authorised individuals granted user rights by a REDCap administrator at York CTU. All data will be kept secure at all times and maintained in accordance with the requirements of GDPR and archived according to GCP regulations.

### Sample size and power calculations

This trial has two co-primary outcomes (4-week average Longitudinal Follow-Up Evaluation (LIFE) [50] depression and mania PSR (Psychiatric Status Ratings). However, the overall symptom severity of LIFE [50] depression is greater than that of mania in this population, as is its standard deviation (SD), and therefore a larger sample size is required for the depression component compared with the mania component [73]. Therefore, sample size is based primarily upon parameters from the depression component. Although the minimally clinically important difference (MCID) in LIFE [50] PSR mania score is somewhat smaller than the MCID for the LIFE [50] depression score, the relative difference will be smaller than that for the SD. Overall, the standardised effect size will be smaller for depression than mania, thus leading to a larger sample size. On the LIFE [50] PSR, our eligible population will score at the higher end of the subthreshold range (3–4) as those scoring 5–6 will confer research diagnostic criteria and therefore not meet the inclusion criteria for the trial. Based on our feasibility trial [74] where participants had a mean baseline BDI-II score of 37.9, participants will tend to be towards the upper end of the subthreshold range. We therefore expect our LIFE [50] depression PSR score to be 3.75. Button [75] reported that a MCID for the BDI-II is around 17–18%. Given that we expected similar sensitivity for the mean LIFE depression PSR scores as would be the case for BDI-II, a difference of around 0.5 points (0.18*[3.75–1] = 0.50 to 2 decimal places) on the PSR for LIFE [50] depression will be considered the minimum in order to indicate that CBT_BAR_ is having an important effect.

Assuming a SD of 1.3 [73], a conservative correlation of 0.4 between baseline and 27-weeks [76] a 2.5% two-sided significance level, and a between group mean difference of 0.5 points in mean 4-week PSR for LIFE [50] depression at 27-weeks, we would require 286 participants with outcome data to achieve 90% power. Inflating the sample size to allow for a conservative 15% attrition (13% in the feasibility trial) [74] requires a target to randomise of 338 participants (approximately 68 per site). This sample size would also provide 98.5% power to detect a more conservative MCID on LIFE [50] PSR for mania, target effect of 0.25 points, estimated within-group SD = 0.5 points [73]. In each case, power will be increased due to the (multiple) correlation between outcome and the full set of explanatory variables adjusted for in the model. A random therapist effect is not accounted for in our sample size calculation as the effect on the BDI-II in our feasibility trial was 0, although the confidence interval was wide. This will be explored within a sensitivity analyses.

For the qualitative component we will recruit two groups of 15–25 people each for individual interviews. One group will be participants from the intervention arm of the trial across all five sites and one group will comprise key stakeholders including health professionals, service providers and commissioners. To build upon the acceptability work in the original BART trial, we will seek a more diverse and inclusive sample (e.g. ethnicity, socioeconomic status, gender) and level of engagement in therapy, including non-responders. The stakeholder groups will include health professionals involved in delivery of CBT_BAR_ and other interventions and services for this population, and referrers (and potential referrers) to the trial from a range of services and across geographical area.

#### Data analyses

Quantitative analyses will be undertaken using the principles of intention-to-treat, where participants are analysed according to the group to which they were randomised, regardless of what treatment they received. Analysis will be undertaken using Stata v18.0 (or later) [45], using two-sides tests at a 2.5% significance level, with 97.5% confidence intervals provided, unless otherwise stated. All quantitative analyses will be pre-planned and included in a Statistical Analysis Plan which will be finalised and approved by the TSC prior to database lock and analysis.

#### Co-primary outcomes

The primary analyses for each of the co-primary outcomes (mean 4-week PSR for LIFE depression score and for LIFE mania score at 27-week follow-up) will use analysis of covariance, with adjustment for the stratification factors (site and BAR group), the baseline PSRs for depression and mania and prior CBT (yes/no). For each outcome, if less than 15% of participants are excluded from the analysis due to missing data, and the differential amount of missing data between the trial arms is less than 10% then complete case analysis will be used. Otherwise, multiple imputation by chained equations (MICE) [77] will be used, assuming that the data are ‘missing at random’. The opposite method will be used as sensitivity analysis. To explore whether BAR group and prior CBT could act as potential moderators of the co-primary outcomes, the analysis will be repeated with the inclusion of interaction term, for each of these variables with treatment arm, separately.

Casual inference methods for mediation [78] will be used to estimate the indirect effect of CBT_BAR_ on each of LIFE mania and LIFE depression scores via the HAPPI total score at the preceding time-point (e.g. 17-week HAPPI for 27-week LIFE scores). A more complex causal model incorporating Behaviours Checklist and Internal State Scale will be investigated using structural equation modelling. As a post-randomisation effect modifier, the impact of the number of CBT_BAR_ sessions attended will be assessed using principal stratification methods; other measures of intervention receipt, including antidepressant medication, will be considered in separate analyses.

#### Secondary outcomes

Secondary outcome measures (including the primary outcome measures at the other follow-up time-points) will be analysed using generalised linear models, with link function appropriate to the type of data. Models will be adjusted in the same way as the primary outcome, using the baseline value of the outcome measure (where applicable). Time to events (transition to first episode (hypo)mania and recovery from BAR symptoms) will be analysed using a Cox proportional hazards model, adjusted for stratification factors. Tests will use a 5% significance level and two-sided 95% CIs will be presented.

#### Sensitivity analysis

The primary analysis will be repeated with the inclusion of random therapist effect in a partially-nested model (clustering by therapist in the CBT_BAR_ + TAU arm but no clustering in the TAU arm). A further sensitivity analysis will use longitudinal mixed-effects model incorporating all follow-up time-points (as factors), fitted using maximum likelihood, accommodating the within-participant correlation over time with an unstructured covariance matrix and including stratification factors and the baseline PSRs for depression and mania as covariates.

#### Economic measures

Data on health status will be collected by the EQ-5D-5L and quality-adjusted life-years (QALYs) will be estimated from the EQ-5D-5L and the utility tariffs recommended by NICE at the time of the analysis. As a comparison, the Recovering Quality of Life (ReQoL-10), will also be collected. This provides an alternative method to estimate QALYs, which is more focused on aspects of mental health, and allows for a comparison between measures. Regarding the service use questionnaire, items of resource use will be multiplied by published national health and social care costs [79].

Analysis will explore associations between NHS and social service use costs and QALY measures and baseline characteristics as well as follow-up outcomes (including SCID LIFE). This will help explain the extent to which service use and QALYs may relate to other outcomes and to identify key baseline characteristics (such as education and employment status). A full economic evaluation is outside of the scope of the research funding, however the data collected in the present study includes sufficient evidence for an economic evaluation to be conducted in future.

#### Qualitative analysis

Interview data will be analysed using reflexive thematic analysis [80, 81], which provides an accessible and flexible approach, resulting in a rich account of qualitative data. We will take a critical realist position, and data will be coded at a manifest level (i.e., analysing only the immediate meaning of participants’ language) to produce an accessible body of coded data from which meaningful thematic representations of participants’ perspectives can be reported. Interviews will be transcribed verbatim and coded dynamically and iteratively within NVivo qualitative data analysis software (Version 11, 2016).

Participant interviews will be analysed to investigate the mechanisms by which the intervention is perceived to operate. Analysis will be conducted by qualitative researchers with lived experience. An inductive approach following the seven steps of Braun and Clarke’s approach [82] will be used whereby researchers will not impose a pre-existing theoretical framework. We will identify and code data that offer relevant information about how participants experience or perceive the intervention to impact on their mood, behaviours and symptoms and draw patterns across participants’ experiences. We will examine the potential barriers and solutions to implementing the intervention into routine care and services that stakeholder participants describe. Regular analysis meetings with the qualitative research team (including interviewers with lived experience) will be key, to further develop emerging thematic and conceptual outputs and ensure that issues related to participant recruitment are transmitted to the teams as quickly as possible.

### Monitoring

#### Trial monitoring

The Trial Management Group will meet monthly to ensure oversight of the trial. Operational meetings will take place more regularly at individual sites. The trial has two independent committees that meet bi-annually to review the trial: the TSC and the DMEC. The DMEC meeting minutes can inform the TSC. The sponsor (Greater Manchester Mental Health NHS Foundation Trust, Ref. x566s) will be responsible for auditing procedures. All protocol amendments are reportable to the funder, sponsor and ethics committee.

#### Harms

Safety will be assessed throughout with rigorous reporting of Serious Adverse Events (SAEs) in line with HRA requirements. Details of the event will be reviewed by the trial management team and chief investigator. Events classified as serious will be reported to sponsor and TSC Chair within 24 h. If classified as “related to the trial” and “unexpected” they will be reported to HRA. All adverse events and serious adverse events will be reviewed by the DMEC and TSC. Following an event, immediate strategies will be put in place to minimise future risk. All the information that is collected about participants will be strictly confidential. However, all participants will be made aware through the Participant Information Sheet and verbally by research assistants and therapists that although their data is strictly confidential, this confidentiality can be broken if they are deemed a risk to themselves or others.

### Patient and Public Involvement (PPI)

We have developed our BAR work in collaboration with service users and carers for over a decade and have researchers with both personal and carer experience as co-investigators. The BART Service User Reference Group (SURG) designed and produced the BART feasibility trial promotional materials, acronym and logo, aided ethics application queries and contributed to the final protocol. All SURG members welcomed this trial, and consultation with individuals and their families led to several ideas being incorporated in this protocol. This included providing technology to SURG members to facilitate attendance at meetings and incorporating an additional phone call for participants at 39 weeks to reduce attrition at follow up. SURG members reviewed and agreed all measures included in this trial, ensuring they would not be overly burdensome. They suggested flexibility in obtaining these measures, such as completion of self-report measures outside of the appointment with the researcher and offering breaks.

Illustrative work of participant’s journeys was created in one of our dissemination and feedback events with participants at the end of the BART feasibility trial. Our SURG group felt strongly that the illustrations should be used in the trial to create an animation for participants and referrers. The BART SURG members felt this would help explain the experiences of service users to referrers and answer questions for potential participants about what to expect if they take part in the study. An additional proposal from our BART SURG was to incorporate families and carers within our PPI work given the needs of this young population and involvement of family members. It was suggested that we have a separate family/carer SURG group enabling specific issues to be discussed separately whilst working concurrently and at times together where either SURG group felt this was important.

### Ethics and dissemination

#### Ethics

The Trial has received Health Research Authority (HRA) approval (IRAS 316335) from the North-West – Greater Manchester West Research Ethics Committee (13th December 2022, 22/NW/0355). All participants will provide written informed consent prior to undertaking research activities. No identifiable information is presented here. Local capacity and capability to deliver the research is provided by the research department at the sponsoring organisation.

#### Dissemination

Dissemination will occur with researchers, staff, service users and PPI representatives. Outputs and results of the trial will be published in open-access peer-reviewed international journals where possible, following the International Committee of Medical Journal Editors guidance [83]. To increase reach and accessibility, results will also be disseminated using PPI input to non-academic audiences via media posts, blogs, newsletters, and written summaries created with the PPI groups.

## Discussion

We anticipate our research could lead to important developments within the NICE Guidelines for Bipolar Disorder (CG185) [24], similar to the recognition and management of those at risk of developing psychosis within the NICE guidelines for Psychosis and Schizophrenia in adults (CG178) [84] and children and young people (CG155) [85]. For the government to meet the targets for the NHS Mental Health Implementation Plan [86] and achieve parity of esteem between mental and physical health for people of all ages, new evidence-based treatments are required. It is imperative that research is undertaken now as we seek to understand how to expand youth service models to widen intake criteria to encompass BD, and those at risk of developing BD, in the aim of reducing symptoms and risk of progression to more severe illness [10, 87, 88]. There is potential for significant savings [1]. With data demonstrating health and economic benefits of early intervention services [12] our trial could provide data for expansion of early intervention for BD including which mechanisms are effective treatment targets in reduction of risk to long-term distressing mood swings. Given our primary research question focuses on mood swings and we do not exclude co-morbid difficulties, our findings likely have broader transdiagnostic applications.

### Trial Status

This paper is in line with approved protocol version 4 02.07.2024. Recruitment to the trial started in February 2023 and will be complete by July 2025.

## Supplementary Information

Supplementary Material 1.

Supplementary Material 2.

## Data Availability

No datasets were generated or analysed during the current study. We will endeavour to ensure the dataset will be made available to other researchers following publication of the study, upon reasonable request and approval by ethical committee.

## Abbreviations

ARMS: At-Risk Mental State
ASRMS: Altman Self Rating Mania Scale
BAR: Bipolar At Risk
BART: Bipolar At Risk Trial
BC: Behaviours Checklist
BCSS: Brief Core Schema Scale
BD: Bipolar Disorder
BDI: Beck Depression Inventory
CALPAS: California Psychotherapy Alliance Scale
CAMHS: Child and Adolescent Mental Health Services
CBT: Cognitive Behavioural Therapy
CBT_BAR_: Cognitive Behavioural Therapy for Bipolar At Risk
CI: Confidence Intervals
CMHT: Community Mental Health Team
CONSORT: Consolidated Standards of Reporting Trials
CRF: Case Report Form
DMEC: Data Management and Ethics Committee
DSM: Diagnostic and Statistical Manual for Mental Disorders
DTQ: Desire Thinking Questionnaire
EDIT: Early Detection and Intervention Team
EIT: Early Intervention Team
EME: Efficacy and Mechanism Evaluation
EQ: Euroqol
FAI: Facilitative Alliance Inventory
GAF: Global Assessment of Functioning Scale
GP: General Practitioners
HAPPI: Hypomanic Positive Predictions Inventory
HRA: Health Research Authority
IAPT: Improving Access to Psychological Therapies
ICM: Integrative Cognitive Model
ISS: Internal States Scores
MCID: Minimally Clinically Important Difference
MCQ-30: Metacognitions Questionnaire 30
MRC: Medical Research Council
NHS FT: National Health Service Foundation Trust
NICE: National Institute for Health and Care Excellence
NIHR: National Institute for Health and Care Research
PANSAM: Positive and Negative Sleep Appraisal Measure
PPI: Patient and Public Involvement
PSQI: Pittsburgh Sleep Quality Index
PSR: Psychiatric Status Ratings
QALYS: Quality-Adjusted Life Years
RCT: Randomised Controlled Trial
REDCaP: Research Electronic Data Capture
REQOL: Recovering Quality of Life
RSQ: Response Style Questionnaire
SAE: Serious Adverse Events
SCID LIFE: Structured Clinical Interview for DSM Disorders Longitudinal Follow-Up Evaluation
S.D.: Standard Deviation
SURG: Service User Reference Group
SOFAS: Social and Occupational Functioning Assessment Scale
SPIRIT: Standard Protocol Items: Recommendations For Interventional Trials
TAU: Treatment As Usual
TIDieR: Template for Intervention Description and Replication
TSC: Trial Steering Committee
YMRS: Young Mania Rating Scale
YTU: York Trials Unit
